# Plummeting Broadcast Storm Problem in Highways by Clustering Vehicles Using Dominating Set and Set Cover

**DOI:** 10.3390/s19092191

**Published:** 2019-05-12

**Authors:** S. Kamakshi, V. S. Shankar Sriram

**Affiliations:** Centre for Information Super Highway (CISH), School of Computing, SASTRA Deemed University, Thanjavur, Tamil Nadu 613401, India; kamakshi@cse.sastra.edu

**Keywords:** vehicular ad-hoc network, intelligent transportation systems, cluster-based information dissemination protocol, message dissemination, connected dominating set, set cover problem

## Abstract

“Vehicular Ad-hoc Networks” (VANETs): As an active research area in the field of wireless sensor networks, they ensure road safety by exchanging alert messages about unexpected events in a decentralized manner. One of the significant challenges in the design of an efficient dissemination protocol for VANETs is the broadcast storm problem, owing to the large number of rebroadcasts. A generic solution to prevent the broadcast storm problem is to cluster the vehicles based on topology, density, distance, speed, or location in such a manner that only a fewer number of vehicles will rebroadcast the alert message to the next group. However, the selection of cluster heads and gateways of the clusters are the key factors that need to be optimized in order to limit the number of rebroadcasts. Hence, to address the aforementioned issues, this paper presents a novel distributed algorithm CDS_SC: Connected Dominating Set and Set Cover for cluster formation that employs a dominating set to choose cluster heads and set covering to select cluster gateways. The CDS_SC is unique among state-of-the-art algorithms, as it relies on local neighborhood information and constructs clusters incrementally. Hence, the proposed method can be implemented in a distributed manner as an event-triggered protocol. Also, the stability of cluster formation is increased along with a reduction in rebroadcasting by allowing a cluster head to be passive when all its cluster members can receive the message from the gateway vehicles. The simulation was carried out in dense, average, and sparse traffic scenarios by varying the number of vehicles injected per second per lane. Besides, the speed of each individual vehicle in each scenario was varied to test the degree of cohesion between vehicles with different speeds. The simulation results confirmed that the proposed algorithm achieved 99% to 100% reachability of alert messages with only 6% to 10% of rebroadcasting vehicles in average and dense traffic scenarios.

## 1. Introduction

The World Health Organization (WHO) report states that due to the presence of road traffic injuries [1], more than 1.3 million people die every year. Delays in perceiving and providing care for those victims increases the severity of injuries; delay of minutes may decide between life and death. Thus VANETs, an imminent part of Intelligent Transportation Systems (ITS), help in disseminating accident notifications immediately after the detection of such event through vehicle-to-vehicle and vehicle-to-road side units (RSUs) communications [2]. Even though RSUs are available in urban regions, we cannot expect the reachability of the RSU for the entire road length especially in highways connecting different states. Similarly, cellular systems are also not available in remote areas. Also, installing the infrastructure needed for supporting cellular technology and RSUs in such remote regions are costlier. Furthermore, there is no support for RSUs or cellular systems, Vehicle-to-Vehicle (V2V) communication through vehicular ad-hoc networks (VANETs) can bridge the gap. VANETs are vehicular communication networks which are instantly and spontaneously established between Dedicated Short-Range Communication-enabled (DSRC) vehicles that do not need any infrastructure support. Hence, a fully distributed algorithm that can operate asynchronously in individual vehicles is necessary to decide on which cluster it has to join, whether it has to act as cluster head or gateway, etc. Road safety applications demand reliable delivery of warning messages to as many vehicles as possible that are approaching towards the zone so that the drivers can take necessary actions at the earliest moment. Though flooding is a simple technique to disseminate the messages to all vehicles, the broadcast storm problem [3] introduces network congestion and delays. Also in VANETs, because of the intermittent connectivity and network partitions due to fast-moving vehicles, providing a stable and reliable communications with high reachability becomes a major challenge [4,5,6]. For reducing the broadcast storm problem, a simple solution is to suppress the rebroadcast of the message to the extent possible without affecting the reachability of the message. Suppression of rebroadcasting can be achieved through selecting a limited number of next forwarders by grouping the vehicles into clusters and selecting the cluster heads and gateways in an efficient manner that ensures reachability. Hence, the research questions at hand are how to group the vehicles into clusters, how to select cluster head vehicle and gateway vehicles in a cluster in an efficient manner such that the number of rebroadcasting vehicles is reduced, but without reducing the reachability of the message.

Vehicles are equipped with sensors for detecting the danger ahead or traffic congestion. Once a vehicle senses an unexpected event, it originates a warning message and disseminates it to the vehicles that are coming towards the zone. To disseminate the information in the wireless medium, broadcasting is a good choice. Among the broadcasting protocols, flooding is a simple network-wide broadcasting protocol [7] that has high reachability. However, it introduces a significant issue called a broadcast storm problem [3], since the vast amount of rebroadcasts results in network congestion. Though the increase in the number of rebroadcasts enhances the reachability of the message to all vehicles of interest, it increases the delay due to higher collisions and retransmissions as well. Hence, the number of messages transferred in the network should be limited to reduce the network delay and congestion. For further rebroadcast, a subset of vehicles that receives the alert message should be selected to control the broadcast storm problem. The selection of such vehicles is achieved by grouping (clustering) the vehicles based on topology, density, distance, speed, location or a combination of these [8,9,10,11,12,13,14,15,16,17,18,19,20,21]. The selection of cluster heads and gateways in cluster-based schemes have a significant impact on the number of rebroadcasts [8,9].

Hence, this research work aims to design a new clustering algorithm that groups the vehicles based on dominating set and set theory to achieve an optimal number of cluster heads and gateways in a fully distributed manner by considering the local neighborhood information and adapting an event-triggered approach that can achieve high reachability with reduced number of rebroadcasts. The significant contributions of the paper are catalogued as follows:

- **To mitigate broadcast storm problem**: Cluster heads are selected based on minimum dominating set and gateways are selected using set cover that minimizes the number of vehicles involved in rebroadcasting the alert message.
- **To increase high reachability**: During low-traffic density to address intermittent connectivity problem, vehicles moving in the opposite direction will carry the live messages and rebroadcast it, when a new vehicle within the zone of relevance moving towards the region of interest whose cluster state is not determined.
- **To ensure high stability of clustering**: The degree of cohesion (DC) of a vehicle among the neighborhood is computed using its relative speed, and the relative distance between vehicles and the vehicle with the highest DC is selected as a cluster head. Also, if a vehicle can hear from a gateway node, but not currently clustered, it is allowed to be un-clustered untill a vehicle with a high degree of cohesion with neighboring vehicles is found.

The proposed algorithm ensures the reachability of the message to all vehicles with a minimum number of rebroadcasts and less message overhead for selection of cluster head and gateway vehicles. The proposed scheme is compared with DTA (Density and Topology-based Approximation) [22] and DWCM (Distributed and Weighted Clustering based on Mobility Metrics) [23] protocols in terms of cluster stability: the average number of state changes per vehicle, cluster density: the average number of members per cluster, cluster head density: the average number of cluster heads per kilometer, cluster state distribution: the percentage of vehicles in each state per kilometer and reachability: the percentage of vehicles that receives the message. The simulation result shows that high reachability of messages was achieved with a minimum number of rebroadcasts.

### Related Works

Since vehicular ad-hoc networks have a lack of a central controller and dynamically changing topology, distributed routing protocols are the most suitable for them. Many researchers have developed scalable and reliable dissemination protocols that spread the message as fast as possible among the vehicles by grouping (clustering) the vehicles based on topology, density, distance, speed, or location for suppressing the rebroadcasts to the extent possible without reducing the reachability. Sun et al. [24] developed two protocols namely, V-TRADE (Vector based Tracking Detection), HV-TRADE (History and Vector based Tracking Detection) that are based on the location of the vehicles. They categorize the vehicles into five classes namely: (i) vehicles that are moving ahead of this vehicle on the same road in the same direction; (ii) vehicles that are coming behind this vehicle on the same road in the same direction; (iii) vehicles that are approaching towards this vehicle on the same road in the opposite direction; (iv) vehicles that are leaving away from this vehicle on the same road in the opposite direction; and (v) vehicles that are moving on other roads. The sender selects zero or more vehicles from each class as a forwarder of the message. In HV-TRADE, the history of the movement of the vehicles is also included in the classification process to provide better precision in the forwarder selection process. Sun et al. [25] designed another protocol named ODAM-C (Optimized Dissemination of Alarm Messages-Capacitor) that selects the relay vehicle based on distance and location. Each vehicle maintains two lists L1 and L0. Upon receiving a packet if the packet is not present in both lists, the packet is placed in the L1 list, and a timer is set for that packet which is inversely proportional to the distance from the sender of the packet. After the timer expires, the packet is forwarded. If the packet received is already present in either L1 or L0, the angle between the current vehicle, original sender, and the current sender is calculated from their location information. If the angle is less than 90°, it will stop the forward of that packet. Otherwise, if the packet is in L1, then stops its timer, deletes from L1, and inserts into L0 and a timer is set. Before the timer in L0 for that packet expires, if it receives duplicate packets, the timer is stopped; otherwise, on expiry of the timer, the packet is forwarded. In all these three protocols the message overhead is higher as each vehicle has to keep track of 1-hop neighborhood information and the packets they have received and forwarded.

Ruiz et al. designed a protocol named BODYF [14,15], a Parameter-less Broadcasting Protocol over Dynamic Forest which constructs a source tree based on a Dynamicity Aware Graph Relabeling System (DAGRS,) model [26]. The next forwarders are the internal nodes of the tree topology formed using the DAGRS model. Nakorn and Rojviboonchai [22] designed a protocol named DTA, with a hybrid approach—a combination of the topology-based and density-based conditions for selecting the vehicles to include in the connected dominating set (CDS). The vehicles with the highest 1-hop neighbor density will become a CDS member. The vehicles that do not have the highest density check for the following three topology-based conditions to overcome network partition. If a vehicle has at least two neighbors that are not directly connected to each other or if it has at least one neighbor that is not covered by its other neighbors, then it becomes a CDS member. If a vehicle has at least one neighbor that is not covered by a pair of gateway vehicles’ neighbors where these two vehicles are neighbors of each other, then it becomes a gateway vehicle. Even though these topology-based conditions avoid network partition problem, they compromise on the optimality of CDS members and result in an unnecessary increase in the number of gateway vehicles. In the proposed method the selection of gateway vehicles is based on set covering problem and it ensures optimality in the number of vehicles selected as gateways. Shi et al. [23] designed a protocol named DWCM (Distributed and Weighted Clustering based on Mobility Metrics) based on the d-hop dominating set. Every vehicle exchanges the mobility information among its d-hop neighbors and calculates its priority based on the relative velocity and relative acceleration with its neighborhood vehicles. Each vehicle maintains two lists, one for holding mobility information of its d-hop neighbors from which it calculates its priority and the other for each d-hop neighbor the id of the maximum priority vehicle among its d-hop neighbors. The vehicle will act as a cluster head if it has the highest priority among its d-hop neighborhood or it has the highest priority among the d-hop neighborhood of its d-hop neighbors. The vehicle will act as a cluster gateway if it can hear from more than one cluster head. This gateway selection has the problem of choosing multiple gateways between the same two cluster heads, which is addressed in the proposed scheme by selecting a minimum number of gateways based on set covering.

Ros et al. [27] developed a fully distributed, reliable broadcasting protocol named ABSM, an Acknowledged Broadcast from Static to Highly Mobile. It combines Connected Dominating Set (CDS) concept with a Neighbor Elimination Scheme (NES). Each vehicle based on the current network topology information calculates CDS and determines whether it belongs to it or not. Upon receipt of a message for the first time, the vehicle initializes two lists: a list R, containing the list of neighbors that are believed to be received that message, and a list N, containing the list of neighbors that are in need of the message. Then the vehicle sets a waiting timeout period. The vehicles that belong to CDS select a shorter timeout period compared to those that do not belong to CDS. During the timeout period, it monitors the message retransmissions from other neighbors and updates the R and N lists. After the timeout expires if the N list is non-empty, then it retransmits the message otherwise it cancels the retransmission of the message. This protocol also suffers from high message overhead in keeping track of the R and N lists.

Vegni et al. [11] designed a protocol named SRB (Selective Reliable Broadcast) an opportunistic broadcast protocol in which the source vehicle selects the next forwarder based on the distance. The clusters are detected based on the distance between each pair of vehicles which are less than a threshold value. The source vehicle elects the furthest vehicle inside each cluster detected as the cluster head and the cluster heads will become the source vehicle for the next contention phase. Since the cluster formation is based only on distance, due to differing speed between vehicles the duration of connectivity among vehicles in the same cluster suffers from frequent disconnections. In our proposed technique the cluster formation is based on distance as well as the relative speed of the neighboring vehicles. Ucar et al. [12] designed a protocol named VMaSC-LTE (Vehicular Multihop algorithm for Stable Clustering–Long Term Evolution(4G)) which is a hybrid protocol that combines IEEE 802.11p and the 4G cellular system technologies for achieving high packet delivery ratio and low latency. The cluster head selection is based on the relative speed of the neighboring vehicles. A vehicle is allowed to join the cluster head directly (1-hop) or indirectly (multi-hop) through the neighboring vehicle that is already a member of the cluster in order to reduce the connection overhead. Dong et al. [13] proposed a new protocol named CRB (cluster-based recursive routing) where the accident vehicle which is the source of the emergency message, initially acts cluster head and selects the farthest cluster member based on the ACK message received from the receivers of that message. This process is repeated recursively by considering the currently selected vehicle as the source.

Khan and Cho [28] developed a protocol called BL-CAST (beaconless broadcast) that addresses both the broadcast storm problem and intermittent connectivity. The forwarding vehicle is selected based on distance. Upon receiving a new message, the vehicle starts the timers, which are inversely proportional to the distance from the sender. Before the expiration of the timer, if the vehicle hears the rebroadcast of the message from a back vehicle, the timer is cancelled, and it will not rebroadcast that message; if the vehicle hears the rebroadcasted message from a front vehicle then it ignores that duplicate message and waits for rebroadcast from the back vehicle. Upon expiry of the timer, if there is no rebroadcast of the message from the following vehicle, it schedules the message for rebroadcast. Due to the wait time before retransmission of the message, the delay increases. But road safety applications demand faster delivery of messages to all the vehicles approaching towards the accident zone so that the drivers can choose an alternate route in order to avoid further collisions and traffic jam in that area. Aadil et al. [29] developed a protocol based on Ant Colony Optimization technique to find an optimal cost tour that optimizes two objective functions, one to minimize the distance between the cluster head and its members and the other to balance the number of cluster members attached to a cluster head in order to avoid depletion of power. But in vehicular networks power is not a primary criterion because they can replenish their power [4,5,6]. The tour is constructed by each ant by selecting a node randomly from the search space as cluster head and tries to add other nodes that are within the transmission of the selected node one by one to the cluster using roulette wheel selection method.

Table 1 summarizes few techniques discussed above including the metrics used for evaluating the performance, their advantages and disadvantages.

## 2. Materials and Methods

### 2.1. Network Architecture

**Figure 1:**
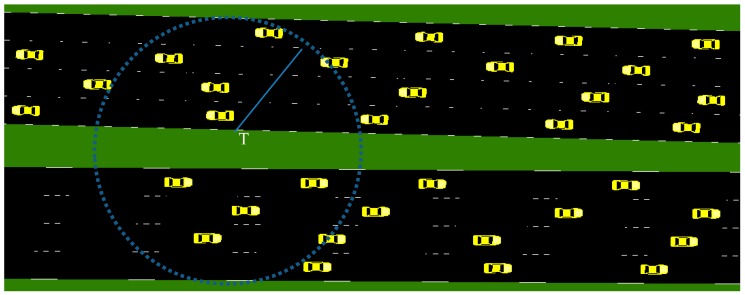
Network model: simulation of highway with 4 lanes in each direction.

The VANETs can be represented as a graph G=(V, E) where the vehicles V represent the vertices, and the communication links E represent the edges. The communication links are assumed to be symmetric. That is two vehicles are connected by an edge if they are in the communication range of each other. SUMO (Simulation of Urban Mobility) is used for generating realistic vehicular traces for a total of 5 km length. Figure 1 shows the snapshot of vehicular traffic in the highways with four lanes in each direction for a road length of 500 m generated by SUMO. Highway roads are assumed as bilateral and have four lanes in each direction. The dotted circle shows the transmission range of the vehicle T. The vehicles need not be synchronised all the time, and each vehicle executes an event triggered protocol in a distributed manner. The protocol operation is given in algorithmic notation in Section 3.6. Different colors are used to represent the current state of the vehicle. The vehicles are initially in “white” color, and their color will be changed to one of the following colors: black (Cluster Head), blue (Cluster Member), red (Gateway), yellow (Candidate Cluster Head), and grey (Candidate Gateway). The proposed method groups vehicles according to their current position and relative mobility of the vehicle and each cluster is identified with the Id of its cluster head. The proposed method chooses a cluster head such that it avoids frequent cluster head changes. Also, it identifies few vehicles among those belonging to more than one cluster as gateways to disseminate the information to all vehicles in the zone of relevance.

### 2.2. Graph Theory

The notations used to describe the proposed algorithm are presented in Table 2.

Thus, the concept of graph theory such as dominating sets and set covers have been employed and the terminologies are given as follows:

**Definition 1** **(Dominating Set).** *Let G=(V, E) be a graph. A vertex v∈V is said to be dominated by another vertex u∈V, if (u, v)∈E. A set D⊆V is called a dominating set of G if for every vertex v∈V is either v is in D, or it is dominated by some vertex u∈D*.

**Definition 2** **(Minimal Dominating Set).** *A dominating set D is said to be a minimal dominating set if none of the proper subsets of D is also a dominating set. The number of vertices in a minimal dominating set of graph G is known as the dominance number γ(G)*.

**Figure 2:**
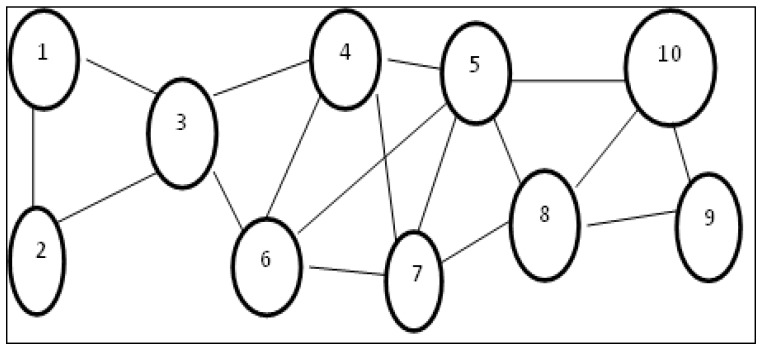
The vertex subsets {1, 6, 8}, {1, 4, 9}, {3, 8}, {3, 5, 9}, and {3, 7, 10} are few dominating sets of G where as {3, 8} is a minimal dominating set. Hence γ(G)=2. The vertex sets {3, 4, 5, 8}, {3, 4, 7, 8}, and {3, 6, 7, 8} are few minimum connected dominating sets Hence $\gamma_{c}\left(G\right)$ = 4.

**Definition 3** **(Minimum Connected Dominating Set).** *If the subgraph induced by dominating set D is connected, then D is said to be a connected dominating set. If no proper subset of D is a connected dominating set, then D is called a minimum connected dominating set and the number of vertices in D is known as connected dominance number, $\gamma_{c}\left(G\right)$. Figure 2 provides a clear distinction between Minimal Dominating Set and Minimum Connected Dominating Set using an example*.

**Definition 4** **(Minimum Set Cover).** *Given a universe U and a family F of subsets of U, a set cover is a subfamily C⊆F of sets whose union is U, the set cover with minimum size is called minimum set cover*.

Consider U = {1, 2, 3, 4, 5, 6, 7, 8, 9, 10, 11, 12}, F = {$S_{1}$, $S_{2}$, $S_{3}$, $S_{4}$, $S_{5}$, $S_{6}$}, where $S_{1}=\left\{1,\;3,\;5,\;6,\;10,\;11\right\}$, $S_{2}=\left\{2,\;5,\;7,\;9,\;12\right\}$, $S_{3}=\left\{1,\;3,\;5,\;9,\;10,\;12\right\}$, $S_{4}=\left\{1,\;4,\;7,\;9\right\}$, $S_{5}=\left\{7,\;8,\;9,\;10,\;11,\;12\right\}$, $S_{6}=\left\{2,\;4,\;6,\;8,\;10,\;12\right\}$ and $C=\left\{S_{1},\;S_{3},\;S_{4},\;S_{6}\right\}$ is a set cover where as $C=\left\{S_{1},\;S_{2},\;S_{6}\right\}$ is a minimum set cover.

Finding the minimum connected dominating set and minimum set cover are NP-Complete problems [32,33]. Hence, a connected dominating set that is close to minimum connected dominating set and the set cover which is close to minimum set cover has been identified. The proposed algorithm aims to disseminate the information to all the vehicles approaching the region of interest with minimum rebroadcast, by clustering the vehicles into groups using the concept of dominating sets and set covers. Each vehicle calculates its degree of cohesion with its neighborhood and the vehicle with a higher degree of cohesion is selected as a cluster head and is included in the dominating set. The vehicles that receive beacons from more than one cluster head will become the candidate gateway. The cluster head receives the set of cluster heads known by all the candidate gateways belonging to its cluster and finds a minimum set cover of these subsets to select the gateways. The set of selected cluster head vehicles and the selected gateway vehicles forms a connected dominating set.

## 3. Proposed Method

### 3.1. Cluster Formation

Each vehicle periodically broadcasts beacon message that includes its Id, speed, and the direction of movement, color, and cluster Id ($C_{id}$). Upon receiving the beacon messages each vehicle “u”, updates its neighbourhood table ($N_{u}$). Initially, the vehicle’s colour is white, and $C_{id}$ is −1. Once it identifies a cluster head in its neighbourhood, its colour becomes blue, and its $C_{id}$ is set to the Id of its cluster head. If it identifies more than one cluster head within its neighborhood, its color becomes grey, and it’s $C_{id}$ includes the Ids’ of those cluster heads. If it cannot identify any cluster head in its transmission range within a limited period and has white/yellow neighbors, its color becomes yellow, and the yellow nodes participate in the cluster head selection process.

### 3.2. Cluster Head Selection

If the cluster head is selected purely based on the relative distance between other vehicles, then due to the varying speed of the vehicles the cluster maintenance becomes tedious. Hence, cluster head and gateway selections are not only based on their relative positions, but also proportionate weighting is given for the speed of the vehicle. This reduces the frequent change in the cluster state. The neighborhood of a vehicle is determined by its relative mobility concerning the speed of the vehicles within its transmission range.

When a vehicle u receives the beacon messages sent by other vehicles moving in the same direction within its transmission range and builds its neighborhood table ($N_{u}$). If the neighbourhood is empty, then it changes its colour to black and becomes the cluster head. Otherwise, it calculates the degree of cohesion (*DC*) as,

(1) $$DC_{u}=\frac{1}{\left|N_{u}\right|}\;\underset{v\in N_{u}}{\sum }\frac{rm_{uv}}{d_{uv}}$$

where $rm_{uv}=\frac{min\left(S_{u},\;S_{v}\right)}{max\left(S_{u},\;S_{v}\right)}$ is the relative mobility of the vehicles u and v, $S_{u}$ is the speed of vehicle *u*, $S_{v}$ is the speed of vehicle v, and $d_{uv}$ is the distance between vehicles u and v. The distance between the vehicles may be approximately calculated by the received signal strength. The vehicles with yellow color, exchange their degree of cohesion within their 2-hop neighborhood. On receipt of the *DC* value from all vehicles with yellow color in its 2-hop neighborhood, the vehicle with the highest *DC* value changes its color from yellow to black. The other white/yellow vehicles upon receiving the updated beacon message from the selected cluster head change their color to blue and set it’s $C_{id}$ to the id of its cluster head.

### 3.3. Gateway Selection

The vehicles that can receive messages from more than one cluster head change its color to grey and it’s $C_{id}$ is set as the concatenation of the Ids of all the cluster heads. The cluster head initiates gateway selection process when it receives the beacon message from grey vehicles. Using the received beacon messages, the cluster head computes the union of all neighboring CHs. All these neighbouring CHs are initially marked as uncovered. The cluster head repeats the following process until all neighboring CHs are covered: For each grey vehicle the cluster head computes the number of still uncovered CHs, and selects the grey vehicle with maximum value. The neighboring CHs that are covered by the currently selected grey vehicle are marked as covered. Once all neighboring CHs are covered the cluster head sends *GW* message consisting of selected gateway vehicles. Upon receiving the *GW* message, the selected vehicles change its colour from grey to red and the unselected vehicles change its color from grey to blue and updates its $C_{\mathrm{id}}$ to the Id of the cluster head with whom its relative mobility ratio is higher.

### 3.4. Reduction of Cluster Head Broadcast

To reduce the number of rebroadcasts and also to reduce the message overhead in the cluster maintenance, the cluster heads are categorized as active or passive. After selection of gateway nodes, each gateway node ‘u’ sends its neighbourhood information ($N_{u}$) to its CH neighbours. Each CH checks if all its cluster members can be covered by one or more of the GW nodes. If so, it becomes a passive cluster head and will not participate in the rebroadcasting of messages. Otherwise, it remains as an active cluster head and will take part in rebroadcasting the messages.

**Figure 3:**
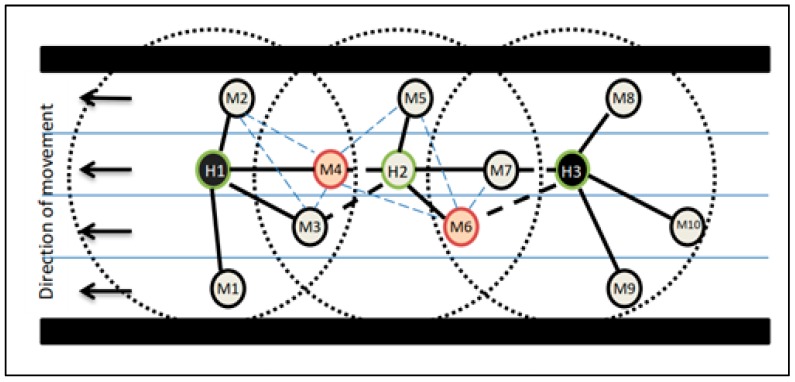
Cluster formation and gateway selection.

Figure 3 depicts a snapshot of the vehicle traffic on a four-lane road. For clarity, the vehicles are shown as solid circles and are labelled. Dotted circles represent the transmission ranges of vehicles H1, H2, and H3. Solid edges are drawn between the cluster head, and its members and dotted black, dark edges are drawn between the cluster head and candidate gateway nodes. The dotted blue thin edges are drawn between the gateway and its members.

After the initial cluster formation and gateway selection, H1, H2, and H3 are selected as cluster heads, and vehicles M4 & M6 are chosen as gateways. Upon receiving the neighborhood table of M4 and M6, the cluster head H2 marks itself as passive since all its cluster members are covered by the gateways. To maintain stability in the network, the cluster head state of H2 is not withdrawn. For example, if H2 withdraws its state from cluster head, then M4 and M6 cannot continue to be a gateway and M5 becomes un-clustered which results in reconstructing the cluster again.

### 3.5. Cluster Maintenance

Whenever a cluster member goes out of range of its cluster head for consecutive three beacon intervals, the vehicle changes its color to white and checks its neighborhood information ($N_{u}$) to determine if there is any other active cluster head available in its range. If so, it becomes a member of that cluster and sets the colour and $C_{id}$ accordingly. Otherwise if it senses any passive cluster head in its transmission range, the vehicle sends a request to that cluster head to trigger it as an active cluster head, and join in that cluster; if there is no cluster head, but a GW node is available in the neighborhood, it remains un-clustered, however still receives messages that are broadcasted by that GW node. If no GW is available, then it initiates the cluster head selection process. If no white neighbours exist in its neighbourhood for a given period, then the node becomes cluster head and the blue neighbours in its range when receiving the beacon from the new cluster head node becomes candidate GW and the GW selection process starts.

**Figure 4:**
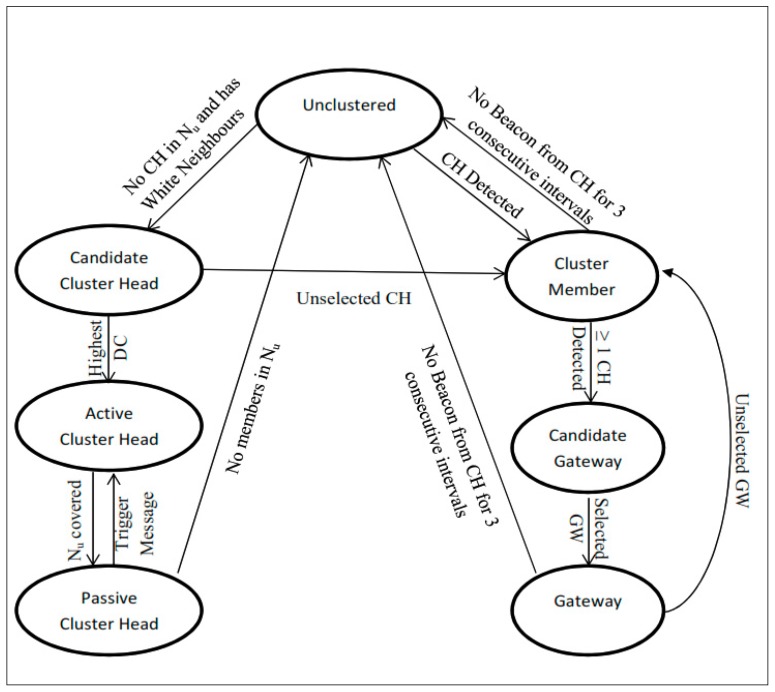
State transition in vehicles.

Figure 4 represents the state transition diagram showing when the state transition is triggered in vehicles during the execution of the proposed algorithm. Initially, all the vehicles are in the “Unclustered” state. If there is any cluster head detected in its neighborhood, it becomes the member of that cluster and transits to “Cluster Member” State. If it can hear from more than one cluster head in its transmission range, then it transits to “Candidate Gateway” state. If there is no cluster head detected in its transmission range, having few more “Unclustered” vehicles then it transits to “Candidate Cluster Head” state. After exchanging DC value among 2-hop neighbors, if it has the highest DC then it transits to “Cluster Head” and other vehicles with lesser DC will transit to “Cluster Member” state. A candidate gateway vehicle upon receiving GW selected message from its cluster head transits to the “Gateway” state and others which are not selected will transit back to the “Cluster Member” state. A cluster member vehicle or gateway vehicle not receiving beacon message for three consecutive intervals will transit back to the “Unclustered” state. Also, a cluster head having no neighbors in its transmission range transits back to the “Unclustered” state.

**Figure 5:**
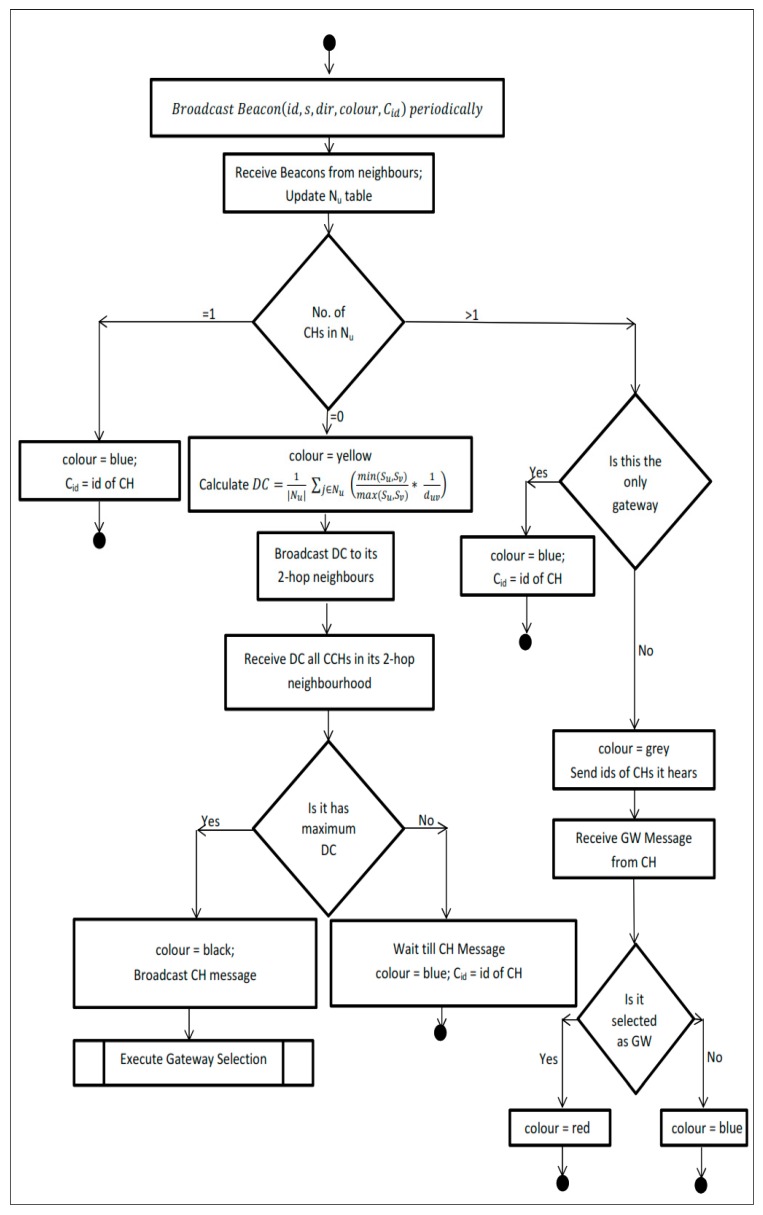
Cluster formation algorithm.

**Figure 6:**
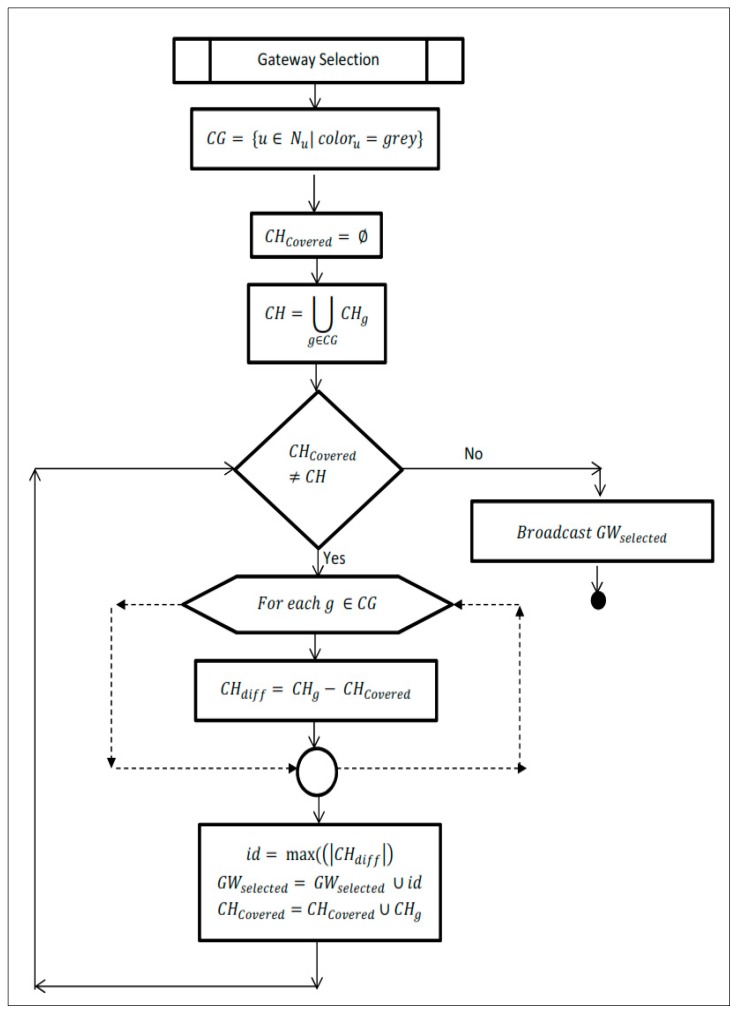
Gateway selection algorithm.

Figure 5 and Figure 6 show the flow chart for cluster formation and gateway selection processes. All vehicles periodically broadcast the beacon message consisting of (id, speed, direction, color, and cluster id). Each vehicle upon receiving the beacon messages from its neighborhood updates its neighbor table $\left(N_{u}\right)$. If there are no cluster heads which exist in its neighborhood $\left(N_{u}\right)$, then it becomes the candidate cluster head and computes the degree of cohesion as per Equation (1) and broadcast it to its 2-hop neighbors. Upon receiving DC from other candidate cluster heads in its 2-hop neighborhood, the vehicle with highest DC becomes the cluster head (CH) and others upon receiving the CH message, become the cluster member. If only one cluster head (CH) is detected in its neighborhood $\left(N_{u}\right)$ then it becomes the member of that cluster. If there more than one CH is detected, then it attaches itself to the CH with highest DC in its transmission range and it becomes a candidate gateway and sends the ids of the CHs it can hear in its transmission range to its CH. Upon receiving the candidate gateway (CGW) messages, the cluster head initiate the Gateway Selection process as described in Section 3.3. Upon receiving the GW Selected message from the CH, the vehicles which are selected by the CH become the GW and others become cluster member.

### 3.6. Pseudocode

The proposed algorithm follows an asynchronous event-driven model. All nodes execute the initialization routine at the beginning. Whenever an event is detected by a node, depending on its current status, it calls the associated routine. The event may be any one of the following events: receiving a beacon message from its neighborhood, missing of beacon message from a known neighbor for a predefined duration of time, Change of its status to Candidate Cluster Head, receiving of message from a CCH in its 2-hop neighbourhood, receiving message from Candidate Gateway by a Cluster Head, or receiving gateway selection message from its Cluster Head node.

#### 3.6.1. Initialization (Executed by All Nodes)

1. colour=white
2. $C_{id}=-1$
3. $Broadcast\;Beacon\left(id,\;s,\;dir,\;colour,\;C_{id}\right)\;periodically$

#### 3.6.2. Node ‘u’, upon Receiving Beacon Message from Node v (Executed by All Nodes)

1. $if\;dir=dir_{v}then$
2. $\;\;\;if\;v\notin N_{u}\;then\;N_{u}=N_{u}\cup \left\{v\right\}$
3. $if\;colour=black\;and\;colour_{v}=grey\;then\;execute$ Section 3.6.5
4. $if\;C_{id}=-1\;and\;colour_{v}=black\;then\;C_{id}=v;\;colour=blue$
5. $else\;if\;colour=blue\;then\;C_{id}=C_{id}+v;\;colour=grey$

#### 3.6.3. Calculate Degree of Cohesion (Executed by All Candidate Cluster Heads)

1. $DC=\frac{1}{\left|N_{u}\right|}\;\sum_{j\in N_{u}}\;\left(\frac{min\left(S_{u},\;S_{v}\right)}{max\left(S_{u},\;S_{v}\right)}*\frac{1}{d_{uv}}\right)$
2. Broadcast DC *up to 2-hop neighbours*

#### 3.6.4. Upon Receiving Messages from Candidate Cluster Heads (CCH) within 2-Hop Neighbors (Executed by Candidate Cluster Heads Only)

1. $\forall v\in CCH\;if\;\max\left(DC_{v}\right)=DC\;then\;color=black;\;C_{id}=id$
2. Broadcast CH message to neighbours

#### 3.6.5. Upon Receiving Neighborhood Information from Candidate Gateways (CG) (Executed by Cluster Heads Only)

1. Add id to CG
2. $Split\;C_{id}\;and\;add\;them\;to\;CH_{g}$

#### 3.6.6. After Receiving Neighborhood Information from all Candidate Gateways (CG) in Its Neighborhood (Executed by Cluster Heads Only)

1. $CH_{Covered}=\emptyset$
2. $CH={⋃}_{g\in CG}CH_{g}$
3. $While\;CH_{Covered}\neq CH$
4.   For each g ∈CG
5. $\;\;\;CH_{diff}=CH_{g}-CH_{Covered}$
6. $\;\;id=\max\left(\left|CH_{diff}\right|\right)$
7. $\;\;GW_{selected}=GW_{selected}\cup id$
8. $\;\;CH_{Covered}=CH_{Covered}\cup CH_{g}$
9. $Broadcast\;GW_{selected}$

#### 3.6.7. Upon Receiving Gateway Selection Message: (Executed by Candidate Gateways only)

1. $if\;id\in GW_{selected}\;then\;color=red\;else\;color=blue$

#### 3.6.8. When a Clustered Node u Did Not Receive a Beacon Message from Its CH for Continuous Three Beacon Intervals: (Executed by All Nodes)

1. color=white
2. $C_{id}=-1$
3. $for\;each\;v\in N_{u}$
4. $\;\;if\;dir=dir_{v}\;then$
5. $\;\;\;if\;color_{v}=black\;then\;C_{id}=v;\;color=blue;and\;exit$
6. $for\;each\;v\in N_{u}$
7. $\;\;if\;dir=\;dir_{v}\;then$
8. $\;\;\;if\;color_{v}=grey\;then\;color=cyan;and\;exit$
9. color=yellow and executeSection 3.6.3 and Section 3.6.4

## 4. Simulation Results and Performance Analysis

### 4.1. Experimental Setup

The proposed method wa simulated in TETCOS NetSim™ V10.2 using SUMO for generating realistic vehicular traffic traces. A highway with four lanes in each direction for 5 km length was considered under simulation. The vehicle speeds varied from 7 m/s to 27 m/s. If the total number of vehicles moving in the same direction in all four lanes on the road per kilometer was less than or equal to 15, then it was considered a sparse traffic scenario, if it was between 16 and 30, then it was considered an average traffic scenario, and if it was between 31 and 50 (and above) it was considered a dense traffic scenario. The performance of the protocol was measured in all three scenarios. The vehicle’s movement was predicted using the Modified Krauss Mobility Model which was implemented in SUMO with some modifications to the original Krauss model. Matlab^®^ was interfaced with TETCOS NetSim™ for implementing the clustering process. The performance of the proposed algorithm was evaluated in terms of the cluster stability, cluster density, cluster head density, cluster state distribution throughput, packet delivery ratio, end-to-end delay, packet collisions ratio, and packet loss ratio. The number of messages transmitted for cluster formation and maintenance was also extensively studied.

### 4.2. Simulation Settings

Table 3 Lists of the simulation parameters. The simulation was run for 2 hours. The transmission range of a vehicle was limited to 250 meters. For the warning messages, the region of interest was considered to be 5 km from the place of incident and period of validity of the message to 30 minutes.

**Figure 7:**
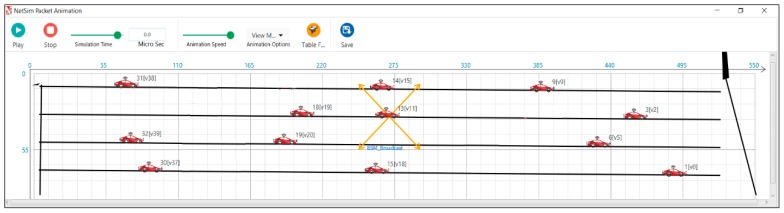
Clustering of vehicles in an average traffic scenario.

**Figure 8:**
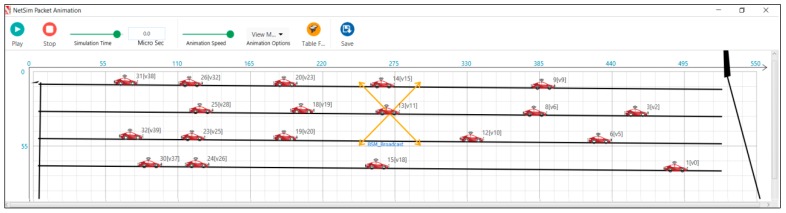
Clustering of vehicles in dense traffic scenario.

**Figure 9:**
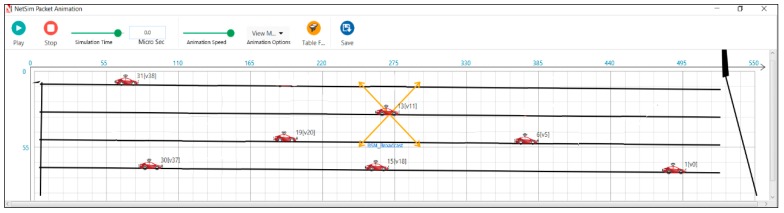
Clustering of vehicles in sparse traffic scenario.

A sample snapshot of the simulation run for a length of the road of 500 m is provided for all three scenarios. Figure 7 depicts the snapshot of the simulation run in an average traffic scenario. Figure 8 depicts the snapshot of the simulation run in a dense traffic scenario. Figure 9 depicts the snapshot of the simulation run in a sparse traffic scenario.

### 4.3. Performance Metrics

The performance of the proposed scheme CDS_SC is evaluated in terms of cluster stability, cluster density, cluster head density, and percentage vehicles acting as a cluster head, cluster member, and gateways. The cluster stability was measured by how many times the color (state) of a vehicle changed during the simulation represented in terms of percentage of vehicles. The cluster density was measured as a number of members belonging to one cluster with the increase in the number of vehicles per kilometer. Cluster head density was measured as the average number of cluster heads per kilometer, cluster state distribution was measured as the percentage of vehicles in each state per kilometer and packet delivery ratio was calculated as the percentage of vehicles that receives the message. End-to-end delay was computed as the average time required for disseminating the alert message from the source vehicle to all the vehicles in the 5 km range approaching towards the region of interest. The packet collision ratio was calculated as the percentage of packets that collided during transmission and the packet loss ratio was calculated as the percentage of packets that were lost during the transmission.

### 4.4. Existing Algorithms Used for Comparison

Our proposed algorithm CDS_SC was compared with recent protocols DTA (Density and Topology-based Approximation) [22] and DWCM (Distributed and Weighted Clustering based on Mobility Metrics) [23]. DTA uses a hybrid approach, a combination of the topology and density-based conditions for computing dominating set members. A vehicle with the highest degree among its neighbors was selected as a cluster head. Gateways were selected as the set of vehicles that had at least two neighbors those were not directly connected to each other or if it had at least one neighbor that was not covered by the other neighbors or if it had at least one neighbor that was not covered by a pair of gateway vehicle’s neighbors where these two vehicles were neighbors of each other. DTA introduces a major bottleneck in sparse and average traffic scenarios due to the selection of a higher number of gateway nodes. On the other hand, DWCM constructed a d-hop dominating set by considering the relative velocity and relative acceleration with the neighborhood. The vehicle will act as a cluster head if it has the highest priority among its d-hop neighborhood. The vehicle will act as a cluster gateway if it can hear from more than one cluster head. DWCM has the problem of choosing multiple gateways between the same cluster heads when the traffic density increases. Hence, the proposed scheme alleviated these problems by selecting a minimum number of gateways based on the set cover.

### 4.5. Comparison Charts

**Figure 10:**
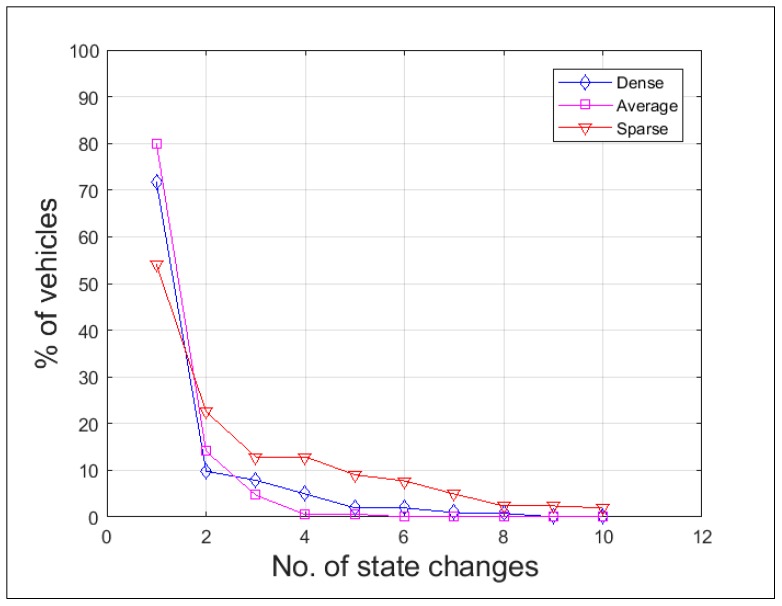
Cluster stability.

For reducing the overhead incurred in the process of cluster formation and maintenance, the formed clusters must be stable. The stability of the clusters is inversely proportional to the number of times a vehicle changes its state. The proposed scheme achieved reduction in the number of state changes by forming the clusters with vehicles having relatively the same speed and selecting the cluster head that had highest degree of cohesion among its 2-hop neighborhood. Figure 10 depicts the cluster stability in all three traffic scenarios. The results showed that almost 80% of the vehicles, once they clustered under one cluster head, remained in the same cluster for a longer duration in an average traffic scenario, during dense traffic it turned out to be 71%, and during sparse traffic it was 54%. During the simulation runs, the cluster state of a vehicle changes on an average of 3 to 4 times and up to a maximum of 10 times.

**Figure 11:**
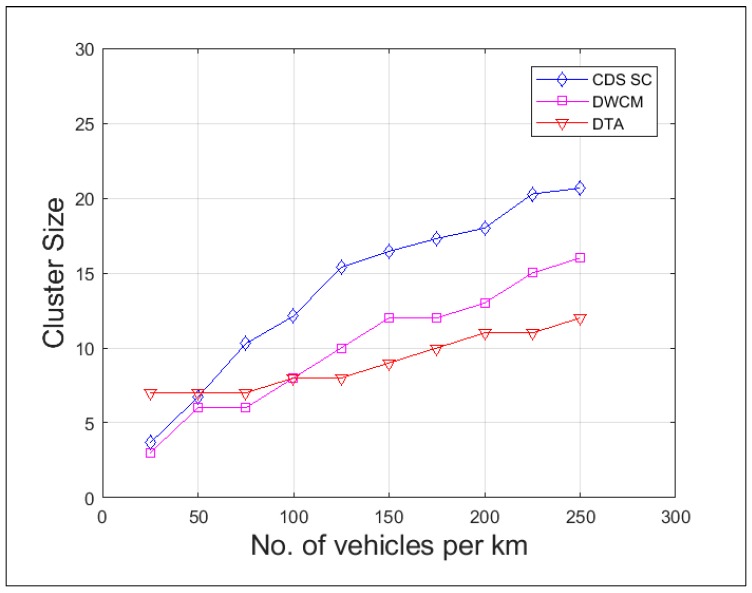
Average number of members per cluster.

The cluster density is measured as the average number of vehicles belonging to the cluster. Figure 11 compares the cluster density of DTA, DWCM, and the proposed scheme CDS_SC. In the proposed scheme the cluster size increases when the number of vehicles increases and thereby the number of clusters decreases. This exhibits the typical behavior of the vehicle movement in a real-time scenario. When the traffic density increases, due to the presence of a higher number of vehicles on the road, the speed of the vehicles within a region is almost the same and their degree of cohesion increases. Hence, the number of vehicles per cluster increases.

**Figure 12:**
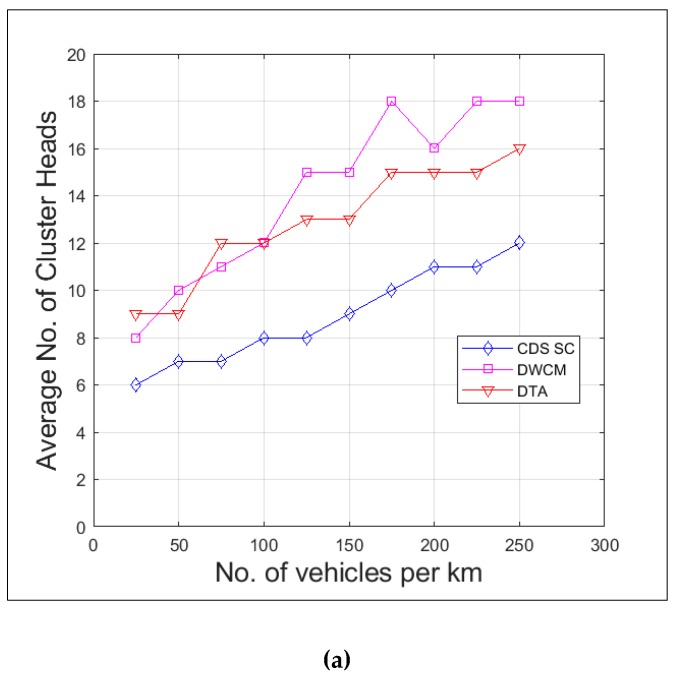

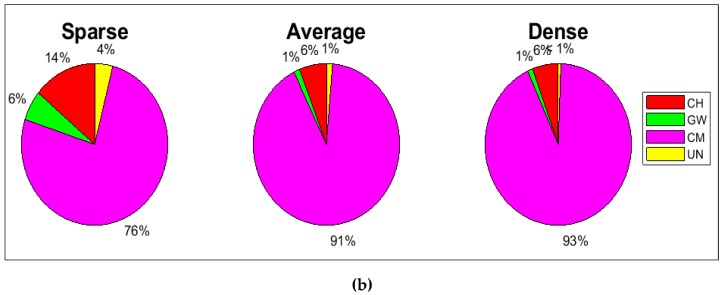
(**a**) Cluster head density (**b**) Cluster state distribution.

The cluster head density is measured as the number of vehicles selected as cluster heads per kilometer. Figure 12a compares the cluster head density of DTA, DWCM, and the proposed protocol CDS_SC. The CDS_SC exhibits a slow increase in the number of cluster heads when the number of vehicles increases. That is when the number of vehicles increases by ten times, the number of cluster heads increases only by twice and this is due to the increase in the cluster size. Figure 12b shows the percentage of vehicles that are identified as cluster heads, cluster members, gateways, and un-clustered in sparse, average, and dense traffic scenarios. The reduction in cluster heads and gateways clearly reveals the achievement of the reduction in the number of rebroadcasting vehicles. In dense and average traffic scenarios the un-clustered vehicles were almost 0% (<1%), and only 6% of vehicles acted as cluster heads and 1% of vehicles acted as gateways. Hence, a total of only 7% of vehicles are rebroadcasting.

**Figure 13:**
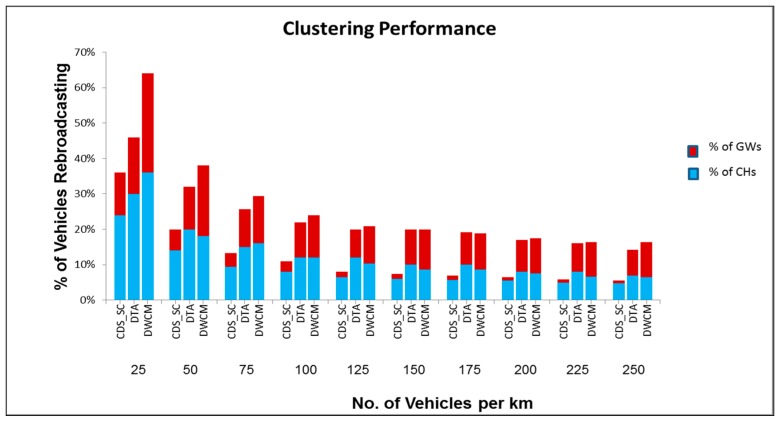
Clustering performance.

Figure 13 compares the percentage of vehicles selected as cluster heads and gateways in DTA, DWCM, and CDS_SC. The proposed scheme outperforms the other two protocols. In CDS_SC, when the network was sparse, nearly 12–36% of the vehicles are rebroadcasting, whereas in dense and average networks only 6% to 10% of the vehicles are rebroadcasting. Compared to DTA and DWCM, the proposed algorithm has one-third reduction in the percentage of vehicles that performs rebroadcasting. This significantly reduces the broadcast storm problem. Due to reduction in rebroadcasting the packet collisions, packet losses, and delay are also reduced. The selection of gateway vehicles based on set cover significantly reduces the number of rebroadcasting vehicles. The proposed algorithm chooses only 4% to 12% of vehicles as gateways, whereas in DTA nearly 7% to 16% of vehicles are chosen as gateways and in DWCM nearly 9% to 28% of vehicles are chosen as gateways.

**Figure 14:**
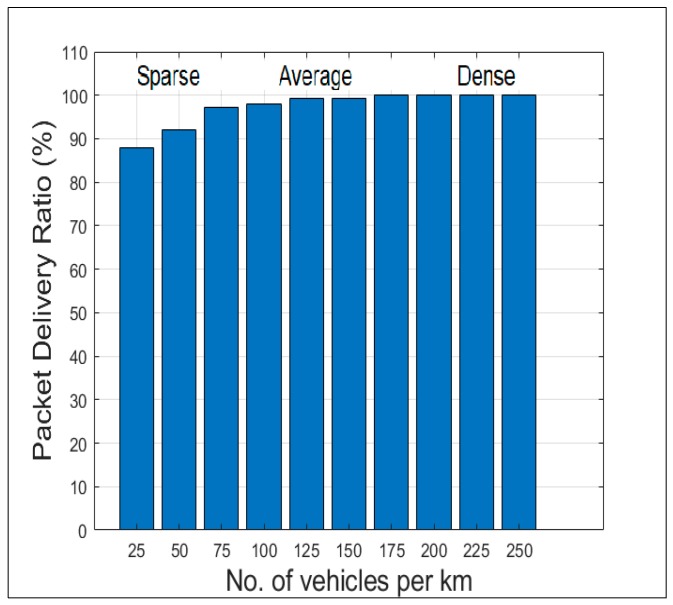
Packet delivery ratio versus vehicle density.

Figure 14 shows the message reachability in the three different scenarios. The packet delivery ratio was calculated as the percentage of vehicles successfully receiving the alert message. In a sparse scenario using a store-and-forward mechanism [31] to overcome intermittent connectivity, 88% to 96% reachability has been achieved. In average and dense scenarios 99% to100% reachability was reached.

**Figure 15:**
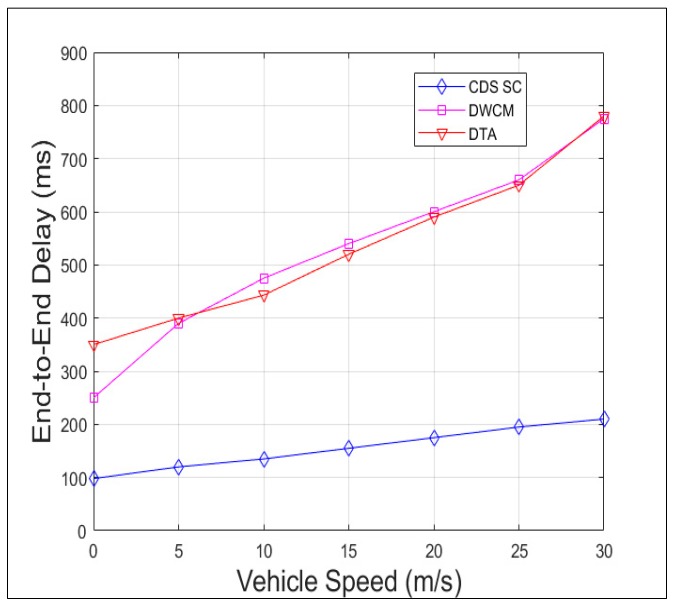
End-to-end delay versus vehicle speed.

**Figure 16:**
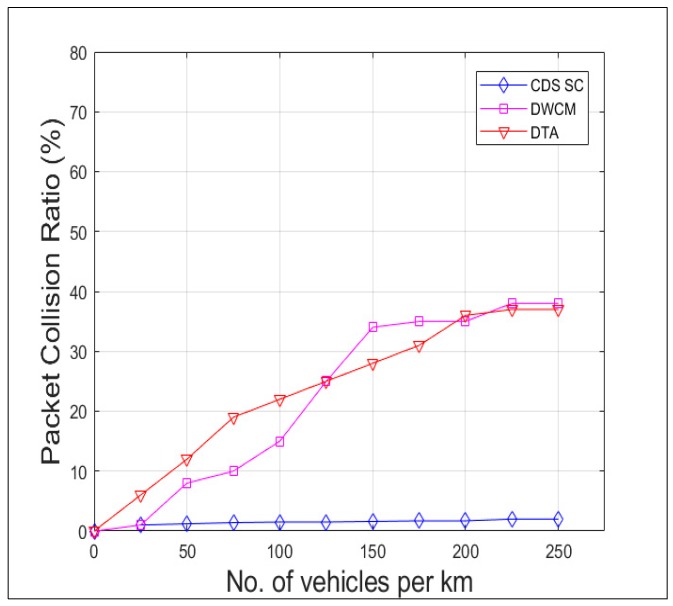
Packet collision ratio versus vehicle density.

**Figure 17:**
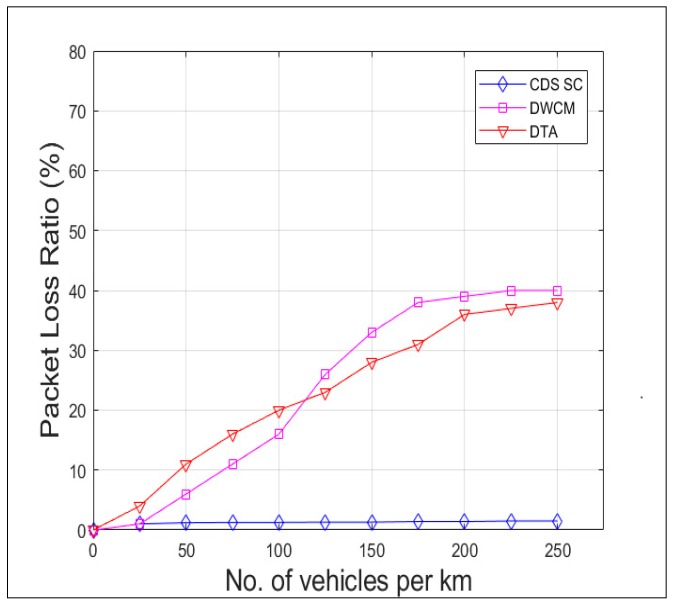
Packet loss ratio versus vehicle density.

Figure 15 compares the delay incurred in receipt of the packet from the source vehicle to all the vehicles in the 5 km range, that is the vehicles in the region of interest by CDS_SC along with DTA and DWCM. The delay is lesser in CDS_SC than the other two because of the faster dissemination due to reduction in broadcast storm and efficient clustering mechanism. Figure 16 and Figure 17 shows the Packet Collision Ratio (PCR) and Packet Loss Ratio (PLR) when the number of vehicles per km increases. Due to the reduction in number of rebroadcasting vehicles both PCR and PLR are negligible and comes around maximum 2% only.

### 4.6. Discussions

The higher stability of the clusters formed by our CDS_SC algorithm ensures the reduction in re-clustering process. Since cluster formation process requires few additional messages like Candidate Cluster Head Message, Candidate Gateway Message, Gateway Selection Message etc., to be exchanged among the neighborhood vehicles, the reduction in re-clustering process significantly reduced the message overhead due to such additional messages. When compared to DTA and DWCM protocols, our CDS_SC had lesser cluster head and gateway vehicles which yielded a predominant decrease in the number of rebroadcasts, thereby alleviating the broadcast storm problem. Ultimately our proposed scheme achieves 99% to 100% message reachability in average traffic scenario with only 6% to 10% of rebroadcasting vehicles.

## 5. Conclusions

Broadcast storm problems and intermittent connectivity are the significant challenges of VANETs. The reduction in number of rebroadcasting vehicles can be achieved through clustering the vehicles into groups and selecting a minimal number of vehicles as next forwarders. A vehicle joins the cluster to which its degree of cohesion is higher which is computed based on the relative mobility among the neighborhood vehicles. The proposed technique provides a solution for reducing the broadcast storm problem by selecting cluster heads based on dominating set and cluster gateways based on the set covering problem. The stability of the cluster head is increased by allowing the state of a vehicle to be un-clustered temporarily if it can receive messages from any other gateway node in its transmission range. The reachability of the messages in the average and dense scenario is approximately 99% to 100%. The predominant decrease in packet loss ratio and packet collision ratio proves the reduction in broadcast storm. As future work, the performance of the proposed technique will be analyzed in a real-time scenario.

## Figures and Tables

**Table 1:** Related Works.

No.	Authors	Protocol Used	Technique Used	Performance Metrics	Advantages	Disadvantages
1.	Sun et al. (2000) [24]	V-TRADE	Distanced-based; select the farthest vehicle in each direction	Bandwidth Utilization, Reachability	Less bandwidth consumption	Intermittent connectivity not addressed
2.	Sun et al. (2000) [24]	HV-TRADE	Distanced-based; select the farthest vehicle in each direction	Bandwidth Utilization, Reachability	High reachability	Intermittent connectivity not addressed
3.	Ruiz et al. (2008) [14]	BODYF	Topology-based; select the internal nodes of the superimposed tree topology	Bandwidth Utilization, Reachability	Less bandwidth consumption, high reachability	The existence of tree topology constructed by some unicast protocol is assumed; Dissemination is slower since the forwarder has to wait for its turn to get the token
4.	Sun et al. (2012) [25]	ODAM-C	Select the farthest vehicle; discard duplicate packets by maintaining lists	Packet Delivery Ratio, End-to-End Delay, Redundancy in transmission	High reachability	Maintenance of timers for each packet received to identify duplicate packets
5.	Khan and Cho, (2014) [28]	BL-CAST	Distance-based; select the farthest vehicle by initialising wait timers which are inversely proportional to the distance from the sender	End-to-End Delay, Message Delivery Ratio, Network Overhead	Less delay, high reachability, less bandwidth consumption	Maintenance of timers for each packet received
6.	Aadil et al. (2016) [29]	ACONET	Distance and speed based; select the forwarder using ant colony optimization	Cluster size, LBF (Load Balancing Factor)	Stable c lustering, optimum number of cluster members per cluster	Proper tuning of parameters (weights assigned to each factor) is necessary
7.	Nakorn and Rojviboonchai (2014) [22]	DTA	Density and topology-based; select dominators of approximate connected dominating set as cluster heads	Reachability, Cluster head Density	High reachability, fast dissemination	High bandwidth consumption
8.	Shi et al. (2016) [23]	DWCM	Topology and speed based; selects the vehicle that has the highest priority among its d-hop neighbours or its d-hop neighbours of d-hop neighbours	Mean cluster head lifetime, Mean re-affiliation time, Number of clusters	Stable clustering	Maintenance of neighbours in d hops and their neighbours; In dense networks, more number of cluster gateways results in unnecessary rebroadcasts
9.	Ros et al. (2012) [27]	ABSM	Location-based; connected dominating set with neighbour elimination scheme	Packet Delivery Ratio, Control Overhead, End-to-End Delay	High reachability, less delay, low overhead, fast dissemination	Maintenance of timers for each packet received; Existence of an efficient algorithm for computing CDS is assumed;
10.	Renê et al. (2017) [30]	Mobility-based scheme for dynamic clustering	Location and speed based; all vehicles within given safer distance will become the CM; The farthest vehicle outside the safer distance in each direction is notified to be a CH	No. of Clusters, CH duration, CM duration, CH change rate, CM connection frequency, Clustering Efficiency	High cluster stability	Temporary CH is required to initiate the clustering process; CH has to maintain a list of its CMs, High bandwidth overhead
11.	Nguyen et al. (2017) [31]	SCF	Distance-based; A vehicle at the edge of a cluster is selected as store-and-carry forwarder if there is no other neighbor vehicle exists within the normalized heading direction between the source and current forwarder	Coverage, Delay, Collision ratio, Message overhead, Efficiency	High reachability	Delay during low-vehicle density; Higher collision ratio during high vehicle density

**Table 2:** Notation.

Notation	Description
Id	Vehicle Id
Dir	Direction of Movement of Vehicle
Colour	State of Vehicle (white, black, blue, red, yellow, grey)
S	Speed of Vehicle
$C_{id}$	Cluster Id
$N_{u}$	Set of all Neighbour Vehicles’ Ids
CH	Cluster Head
GW	Gateway
CCH	Candidate Cluster Head
CG	Candidate Gateway
$DC_{u}$	Degree of Cohesion of Vehicle u
$rm_{uv}$	Relative Mobility between Vehicle u and Vehicle v
$d_{uv}$	Distance between Vehicle u and Vehicle v

**Table 3:** Simulation parameters.

Parameter	Value
Total No. of vehicles injected	1000
No. of lanes per direction	4
No. of vehicles per km per direction	0–15 (Sparse), 16–30 (Average), 31–50 (Dense)
Beacon interval	100 ms (Sparse), 150 ms (Average), 300 ms (Dense)
Transmission range	250 m
MAC Protocol standard	IEEE 802.11p
Propagation model	Two-Ray Ground
Vehicle speed	7 m/s to 27 m/s
Simulation time	2 h
Lifetime for message	30 min
Region of interest	5 km from the place of incident
